# Voluntary Wheel Running Reduces Vesicle Development in an Endometriosis Animal Model Through Modulation of Immune Parameters

**DOI:** 10.3389/frph.2021.826541

**Published:** 2022-01-26

**Authors:** Caroline B. Appleyard, Myrella L. Cruz, Johnathan Velazquez-Cruz, Raquel M. Rivera-Mendez, Juan G. Jimenez-Garcia, Luis A. Rivera, Maria del Mar Mendez-Casillas, Idhaliz Flores, Layla Al-Nakkash, Gladys Chompre

**Affiliations:** ^1^Department of Basic Sciences, Ponce Research Institute, Ponce Health Sciences University-Medical School, Ponce, Puerto Rico; ^2^Biology Department, Pontifical Catholic University of Puerto Rico, Ponce, Puerto Rico; ^3^Department of Biology, University of Puerto Rico at Ponce, Ponce, Puerto Rico; ^4^Department of Physiology, Midwestern University, Glendale, AZ, United States

**Keywords:** endometriosis, exercise, fat, female, fractalkine, muscle, rat, running

## Abstract

**Introduction:**

Endometriosis is a chronic gynecological disorder characterized by the growth of endometrial glands and stroma outside the endometrial cavity producing inflammation and pain. Previously we demonstrated that modulation of the hypothalamic pituitary adrenal (HPA) axis exacerbates the development and severity of this condition. A physically active lifestyle has been shown to confer health benefits in many chronic conditions by potentially acting as a stress buffer, thus we hypothesized that voluntary physical exercise can “realign/reset” the HPA axis resulting in reduced endometriosis symptoms in an animal model.

**Methods:**

Endometriosis was induced in female Sprague Dawley rats by implanting uterine tissue next to the intestinal mesentery on day 0. Sham controls received sutures only. One group of endometriosis animals had access to a running wheel for 2 weeks prior to endometriosis induction until time of sacrifice at day 60. Sham and endometriosis controls received no exercise. All animals were examined for developed vesicles which were collected and measured. Uterine tissue was analyzed for cellular infiltration. Brain, liver, spleen, adrenal glands, leg muscles, and fat were collected, along with peritoneal fluid and blood.

**Results:**

Endometriosis animals developed vesicles in 86.96% of the implants with significantly increased mesenteric fat compared to sham (*p* < 0.05). Exposure to exercise significantly decreased the size (*p* < 0.01) and number (*p* < 0.05) of vesicles that developed, as well as the mesenteric fat (*p* < 0.01). Exercised animals had higher levels of lactoferrin in peritoneal fluid, and decreased serum fractalkine and leptin. Exercise significantly increased estrogen alpha receptor expression levels (*p* < 0.01), while significantly decreasing estrogen receptor beta expression (*p* < 0.01) and macrophage infiltration (*p* < 0.05) in vesicles compared to non- exercised animals.

**Conclusions:**

Our results suggest that voluntary physical activity might protect against endometriosis and alleviate the associated inflammation *via* immune modulation of the HPA axis. This offers the potential for further exploration of exercise as a complementary therapy in endometriosis patients.

## Introduction

It is generally accepted that a physically active lifestyle confers many health benefits and that exercise can act as a stress buffer with beneficial effects in many chronic conditions, including diabetes and inflammatory bowel disease (1, 2). Exercise is thought to reduce stress *via* suppression of the immune system, hypothalamic-pituitary-axis (HPA) regulation (3, 4), and possibly changes in the microflora (5). Patients with endometriosis, a complex chronic gynecological disease characterized by ovarian cysts, peritoneal inflammation, infertility, and pelvic pain (6), suffer with high levels of stress from dealing with the negative impact of their symptoms in all aspects of life (7).

In several case-control studies regular high-intensity (aerobic) exercise has been associated with a reduction in risk for endometriosis (40–80%) compared to women who didn't exercise regularly (8, 9). Moreover, a prospective study also showed an inverse association between total recreational activity and incidence rates of laparoscopically confirmed endometriosis (10). There is also evidence that physical interventions can reduce perceived stress and improve physical conditioning in women with endometriosis *via* normalization of HPA responses (11). However, until now no experimental data exist regarding the impact of exercise as an intervention on the course of endometriosis (12). Further, a recent systematic review and meta-analysis of the literature concluded that physical activity might reduce the risk of endometriosis, but that there are too many confounding factors necessitating the need for more controlled investigation (13).

Voluntary wheel running in rats has been found to cause positive alterations in their immunological, neurochemical, and behavioral responses to stress (14). It can prevent learned helplessness and depressive behaviors (15) and can also attenuate neuropathic pain (16) suggesting the potential to alleviate endometriosis symptoms. The overall aim of this study was therefore to subject rats with experimental endometriosis to a voluntary exercise protocol under controlled conditions, examining the impact on disease progression and inflammation.

## Methods

### Animals

All animal studies were approved by the Institutional Animal Care and Use Committee (IACUC) at Ponce Health Sciences University (PHSU). Female Sprague Dawley rats (Animal Research Facilities, PHSU, PR) of ~2 months old weighing ~180–200 g were randomized to one of three groups: Sham-No exercise (*n* = 9), Endo-No exercise (*n* = 10), or Endo-Exercise (*n* = 10). Animals were housed individually in a room maintained at 23°C with the light/dark cycle alternating every 12 h. Rats were fed with standard laboratory chow *ad libitum*. Weight was monitored twice a week and the experiments were performed at the same time of day to minimize the influence of circadian rhythms.

**Figure 1:**
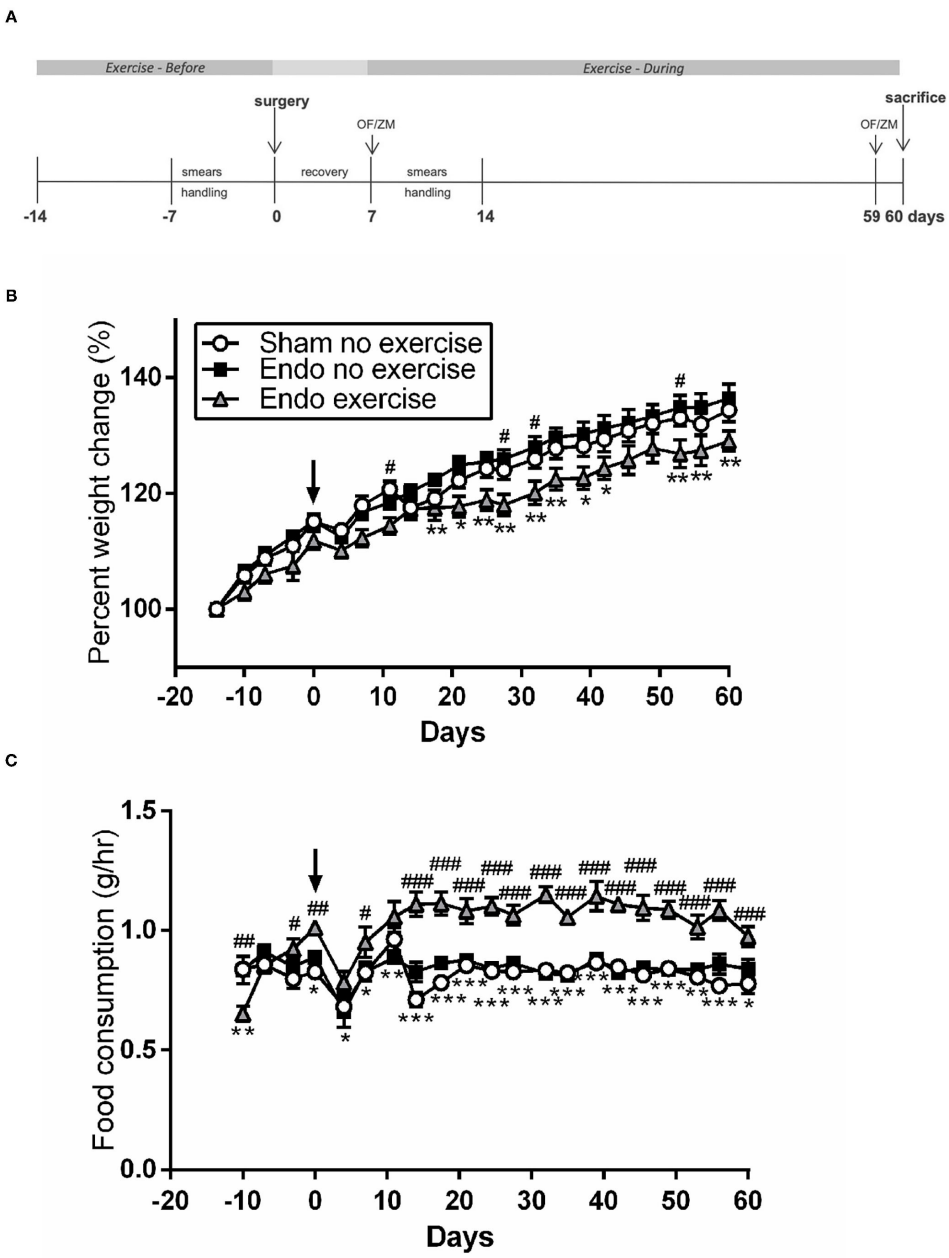
Exercised animals gained less weight while eating more. **(A)** Exercised animals had voluntary access to a running wheel from prior to endometriosis induction until time of sacrifice (OF, open field; ZM, zero maze). **(B)** Weight change throughout the study. **(C)** Food consumption comparison between treatment groups (*n* = 9–10/group ± sem; ^*^p < 0.05, ^**^p < 0.01, ^***^p < 0.001 Endo-No exercise vs. Endo-Exercise; #*p* < 0.05, ##*p* < 0.01, ###*p* < 0.001 Sham-No exercise vs. Endo-Exercise; arrow represents time of surgery).

Endometriosis (Endo) was surgically induced as previously described by suturing autologous uterine horn tissue with the serosal surface next to the mesenteric vessels of the small intestine with the endometrial surface exposed to the peritoneum (17–19). Cytological analysis of smears was carried out before and after surgery (Figure 1A) to verify reproductive cyclicity and to allow for the interpretation of any effects of the stage of estrous cycle on the experimental results.

### Exercise Protocol

Endo-Exercise group animals had free access to a running wheel. These were unlocked for voluntary exercise from day -14 (2 weeks prior to endometriosis induction) until time of sacrifice at day 60 (Figure 1A). All animals were singly housed, where Endo-Exercise rats had running wheel cages (which are slightly larger to accommodate the wheels) with essentially the same free space (volume) as that found in Sham-No exercise and Endo-No Exercise groups which were housed in regular cages. All running wheels had a sensor (counter) connected to an interface (Scurry Interface) to record wheel rotations/min, # running bouts, speed and duration per bout. Cumulative distance (meters), count (revolution), average speed (m/min), and exercise intensity of all Endo-Exercise animals was collected, graphed and analyzed using the Scurry Activity System program to measure exercise parameters. The Scurry Activity program updated the collected data every 3 s (Lafayette Instruments, IN, USA). All animals met the minimum exclusion of 2 km/day (20).

### Behavior Tests (Anxiety)

An open field (OF) behavioral test was administered to the animals 7 days after surgery (day 0) and then again 1 day prior to sacrifice (day 59) to assess behavioral changes related to anxiety. The exploratory and locomotor activity of each rat in an open well-lit arena was measured using Anymaze software (Stoelting), a video tracking system designed to automate testing to track the center point of the rat body for 5 min. On each of the testing days this was followed by use of an elevated zero maze (ZM) 10 min after the open field test finished to assess anxiety related behavior. The elevated zero maze consisted of two open zones and two enclosed dark zones and it was situated in the center of an isolated room with dim illumination. Animals were placed in the elevated zero maze facing a closed zone. Exploratory and locomotor activity of each rat in the open and closed zones were recorded and measured for 5 min using Anymaze software (Stoelting). In each test increased time in the open area/arm indicates lower anxiety.

### Collection of Samples

Blood samples were collected by cardia venipuncture at time of sacrifice. Serum corticosterone levels were determined using an ELISA kit (USA IB79175, IBL-America, Minnesota) according to the manufacturer's instructions. Serum was assayed for cytokine levels using MILLIPLEX MAP Rat Cytokine/Chemokine Magnetic Bead Panel (Cat# RECYTMAG-65K, Millipore). Median fluorescent intensity (MFI) was analyzed and the analyte concentrations were calculated according to standard curve.

All animals had a cytological smear taken, a laparotomy was performed, and after collecting peritoneal fluid by aseptic aspiration the peritoneal cavity was systematically examined for the presence of the implants and the original sutures. Peritoneal fluid was analyzed for lactoferrin levels by ELISA.

The vesicles were identified, and their longest length and width measured with a digital caliper to calculate vesicle area and assign them a score (0–5) as described previously (21). After weighing they were processed for paraffin embedding or frozen whole and stored at −80°C. Samples of uterus were frozen for MPO analysis. The mesenteric fat was removed from the abdominal cavity and weighed; samples were processed for paraffin embedding or frozen. Gastrocnemius and soleus muscles were dissected, removed, weighed, and stored at −80°C. Colon, liver, spleen, jejunum, and adrenal glands were also removed and stored for future follow up studies.

### Mast Cells

Tissue segments (vesicles and mesenteric fat) were fixed in 10% formalin, processed by routine histological techniques, and mounted on glass slides then stained with Toluidine blue to visualize mast cells as previously described (17). The numbers of mast cells in five randomly selected high-power fields were counted by two individual observers (JJ-G and RR-M) and the mean number of mast cells per field calculated.

### Immunofluorescence

Sections from mesenteric fat and vesicle tissue were double labeled with the macrophage marker CD68 and either FKN, CX_3_CR1, or ERβ. Tissue sections were deparaffinized using Xylene Substitute and rehydrated in a series of steps, then boiled for 40 min in Citrate EDTA buffer with a pH = 6 at 95° C. Protein block was performed using Anti-Goat serum. PBS washes were done between steps. Tissue sections were double labeled and incubated overnight with CD68 (Bio Rad #MCA341R; 1:50) and FKN (Abcam #ab25088; 1:1,000), CXC3R1 (Abcam #ab8021; 1:100), or ERβ (Invitrogen #PA1311; 1:500). The next day sections were incubated with secondary antibodies (A11029, Alexa Fluor 488 goat anti-mouse) and (A21428, Alexa fluor 555 goat anti-rabbit) for 30 min, after washing with PBS. DAPI (Nuc Blue Fixed cells stain probe reagent #R37606, Invitrogen) was added and incubated for 5 min. After a PBS wash, the coverslips were mounted using the Prolong Gold Antifade Reagent (Cat#P36934, Invitrogen). Images were obtained using an Olympus BX-60 equipped with X-cite 120Q lamp and photographed with a Nikon DS-FI1 camera. Representative images were taken at 100x, at the same time using the same illumination parameters. FKN, CX_3_CR1, ERβ, and CD-68 expressing cells per high power field (HPF) were analyzed by an observer using Image J software.

### Immunohistochemistry

Vesicle and mesenteric fat tissue sections were deparaffinized in xylene substitute for 30 min and rehydrated in a descending series of alcohols to water. Tissue sections were exposed to 3% Hydrogen peroxide for 15 min. After washing with PBS, antigen retrieval was done in 0.01 M Citrate EDTA buffer (pH = 6) at 95–99°C for 40 min. Tissues were washed with distilled water in two intervals for 2 min each, followed by a PBS wash for 5 min. Protein block was performed using normal goat serum for 15 min (Cat#HK112-9KE, BioGenex), followed by an overnight incubation with ERα primary antibody (1:40; Cat#MA513304, Invitrogen). A negative control with PBS instead of primary antibody was run in each slide. Next day, slides were washed with PBS for 5 min. A Multi-Link was used as the secondary antibody for 20 min, followed by PBS wash for 5 min. The slides were placed in the Streptavidin Peroxidase for 20 min (Super Sensitive Link-Label IHC Detection System, Cat#LP000-UCLE, BioGenex, San Ramon, CA), followed by PBS wash for 5 min. For development, one drop of 3,3′ Diaminobencidine (DAB) (Cat#HK542-XAKE, BioGenex, San Ramon, CA) was used on each tissue and the exposure was monitored for 45 seconds under a light microscope. Then, the slides were dipped in distilled water, washed with running water for 5 min, dehydrated through graded alcohol, cleared with xylene and mounted with Cytoseal 60 (Cat#8310-4, Thermo Scientific). Two representative HPF per tissue were photographed using an Olympus BX-60 and a Nikon DS-FI1 camera and analyzed blindly for ERα expression by an observer using Image J software.

### Adipocyte Assessment

Hematoxylin and eosin staining was performed on collected white adipose tissues to measure size. Photographs from 2 high power fields were blindly assessed by MM-C using ImageJ software (NIH, USA) to measure the length and width of 10 randomly selected adipocytes per photo, to calculate and average μm^2^ per animal.

### Myeloperoxidase Analysis

Tissue myeloperoxidase (MPO) activity was determined in 100 mg of flash-frozen uterine samples as an index of granulocyte infiltration as previously described (21).

### ER mRNA Levels

Thirty milligram of frozen mesenteric fat was placed into 700 μl QIAzol Lysis Reagent in 1.5 ml microcentrifuge tubes for disruption and homogenization. RNA was isolated using the AllPrep DNA/RNA/Protein Mini Kit (Cat# 800104, Qiagen) according to manufacturer's instructions. After mesenteric fat extraction, RNA concentration and integrity was verified using Nanodrop 200 (Thermofisher). mRNA was converted to complementary DNA using iScript cDNA synthesis kit (Cat#1708891, Bio-Rad). Realtime Polymerase reaction was performed using iQ SYBR Green Supermix (Cat#1708882, Bio-Rad) and primers: ERα (PPR44939B), ERβ (PPR48980A), and beta actin (PPR06570C as internal control, Qiagen) following the guidelines from the manufacturer. Data was reported as a fold change or mRNA levels using the equation 2^−ΔCT^.

### Statistical Analysis

Data was analyzed by using GraphPad Instat version 3.0 (GraphPad Software, San Diego, CA) and GraphPad Prism. A *p* < 0.05 was considered to represent a statistical significance difference. The mean difference ± the standard error to the mean (S.E.M.) was used to assess the differences among treatment groups. Values more than 2 SD from the mean were excluded as outliers. In order to assess the statistical significance of the mean differences, a parametric one-way ANOVA was used for normally distributed variables, using the Student-Newman-Keuls. A non-parametric Kruskal-Wallis *H*-test was used for not normally distributed variables (development of vesicles), and the Mann-Whitney *U*-test was used for the *post-hoc* pairwise contrasts after taking into account the accumulation of type I error. A two-way ANOVA was used to analyse the differences in weight change and food consumption at different time points.

## Results

### Exercised Animals Gained Less Weight While Eating More

There were no significant differences in weight between treatment groups at the beginning of the protocol (average 196.62 ± 2.00 g). Animals in the exercise group tended to weigh less at time of surgery, but this did not reach significance. During the protocol all animals gained weight, however the endometriosis animals exposed to exercise gained significantly less weight at various time points than non-exercised (*p* < 0.01; Figure 1B), while having a significantly higher food consumption after surgery (*p* < 0.001; Figure 1C). By the time of sacrifice the Endo-Exercise animals weighed significantly less (253.00 ± 4.12 g) than the Endo-No exercise (271.20 ± 5.70 g; *p* < 0.05) but were comparable in weight to the Sham-No exercise (265.22 ± 3.90 g). No significant differences in anxiety behavior or corticosterone levels were noted among groups (Supplementary Figures 1A–E).

### Voluntary Wheel Running Decreased Vesicle Size

**Figure 2:**
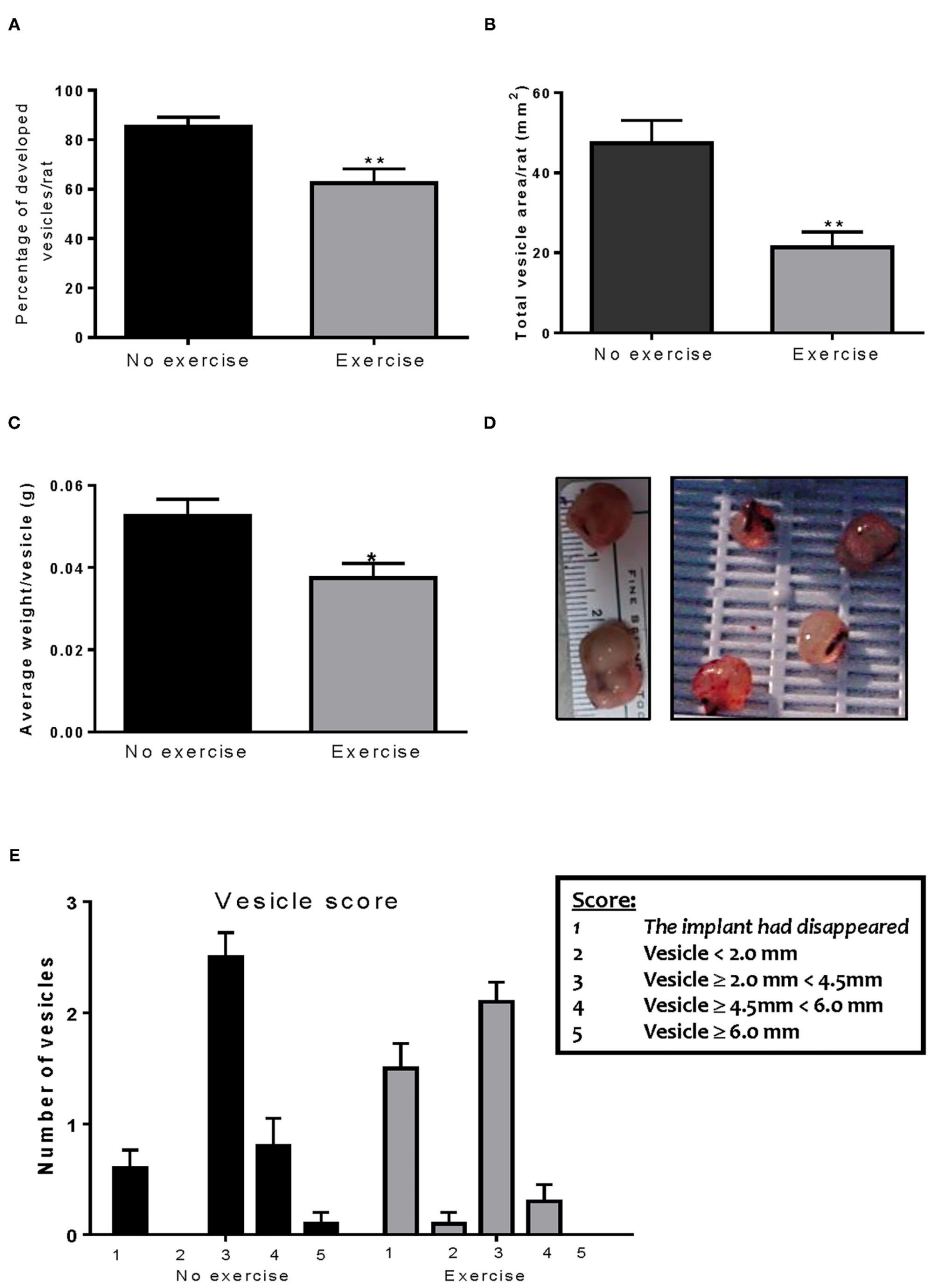
Voluntary wheel running decreases implant size. **(A)** Exercise significantly decreased the percent of vesicles which developed in the endometriosis animals. **(B)** The Endo-Exercise animals had vesicles of a smaller area, which **(C)** weighed less than those found in the sedentary group. **(D)** Representative photos of lesions collected. **(E)** The Endo-Exercise animals developed significantly less vesicles and which were of a smaller size than the non-exercised group (*n* = 10 animals/group ± sem ^*^p < 0.5, ^**^ *p* < 0.01).

After the animals were sacrificed, classification of the vesicles was performed as described previously. Endo-No exercise animals developed vesicles in 85% of the implants (Figure 2A) with an average total area of 47.45 ± 5.70 mm^2^ (Figure 2B) and average vesicle weight of 0.052 ± 0.004 g (Figures 2C,D). Exposure to exercise significantly decreased the number of vesicles that developed (62.5%; *p* < 0.01), as well as their size (21.31 ± 3.86 mm^2^, *p* < 0.01) and weight (0.037 ± 0.004 g, *p* < 0.05). None of the exercise animals developed vesicles which were ≥6.0 mm (Figure 2E). As expected, no vesicles were found in the Sham-No exercise. We found no significant impact of the stress protocol on reproductive cycle length or stage (data not shown).

### Distance and Speed Inversely Correlated With Number of Developed Vesicles

**Figure 3:**
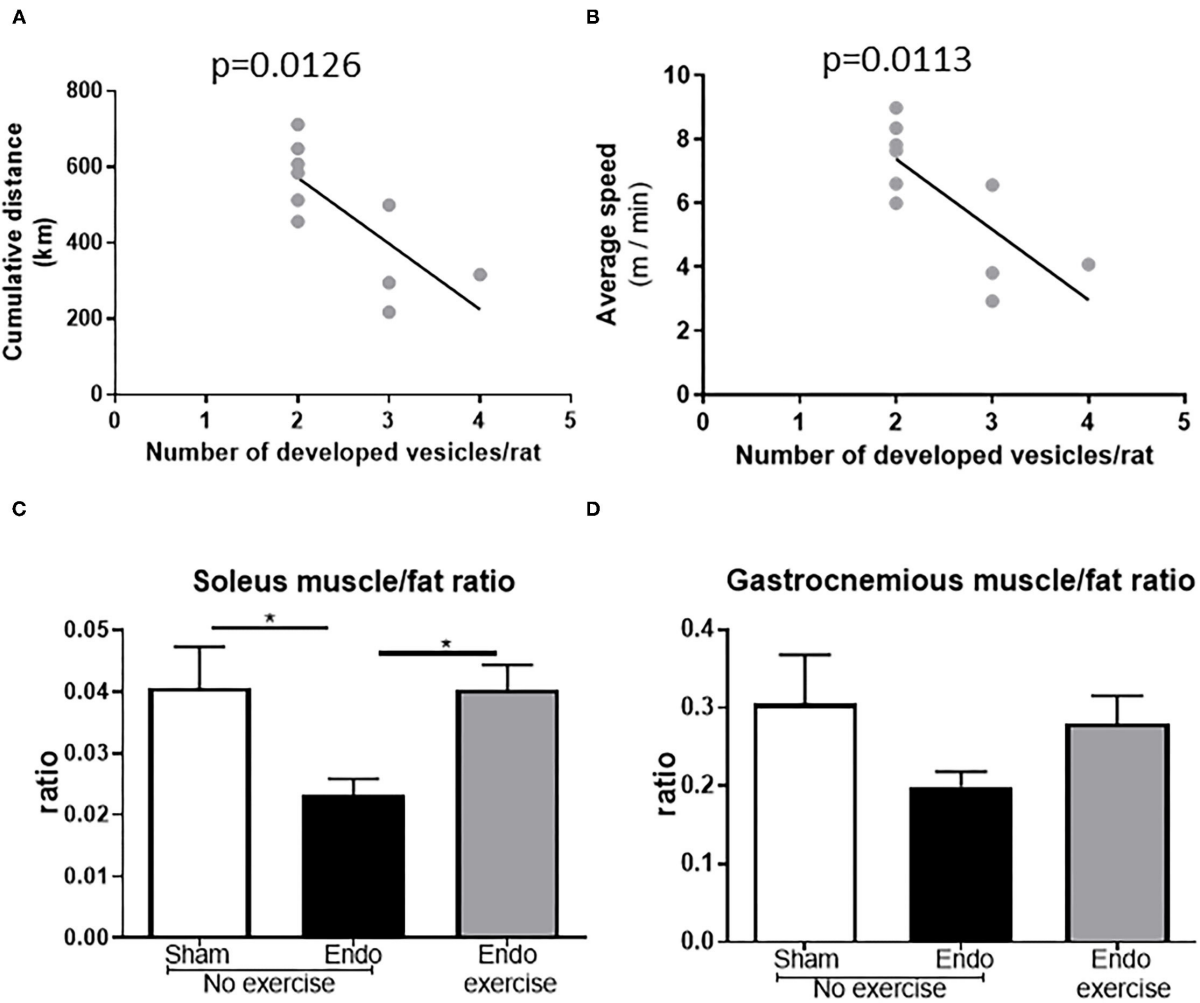
Running parameters and muscles. **(A)** Endo-Exercise animals with higher cumulative distance between days 7 and 60 of the study protocol and **(B)** higher average speeds, developed significantly less vesicles. **(C)** Soleus muscle/fat and **(D)** gastrocnemius muscle/fat ratios increased in the Endo-Exercise animals when compared to the Endo-No exercise animals (*n* = 9–10/group ± sem; ^*^ *p* < 0.05).

All animals adapted to the running wheels quickly and met the minimum exclusion of 2 km/day within 5 days. Distance and speed in Endo-Exercise group were inversely correlated with number of developed vesicles per rat (*p* < 0.05; Figures 3A,B). The muscle/fat ratio was increased in the Endo-exercise group when compared to the Endo-No exercise group (*p* < 0.05), indicating differences were due to the presence of less fat rather than differences in muscle weight following exercise (Figures 3C,D). There was no significant difference in weight of the adrenal glands when normalized to body weight (Supplementary Figure 2A). No significant differences in neutrophil infiltration as assessed by myeloperoxidase measurement was found between groups in the uterus (Supplementary Figure 2B).

### Exercise Decreased Mesenteric Fat

**Figure 4:**
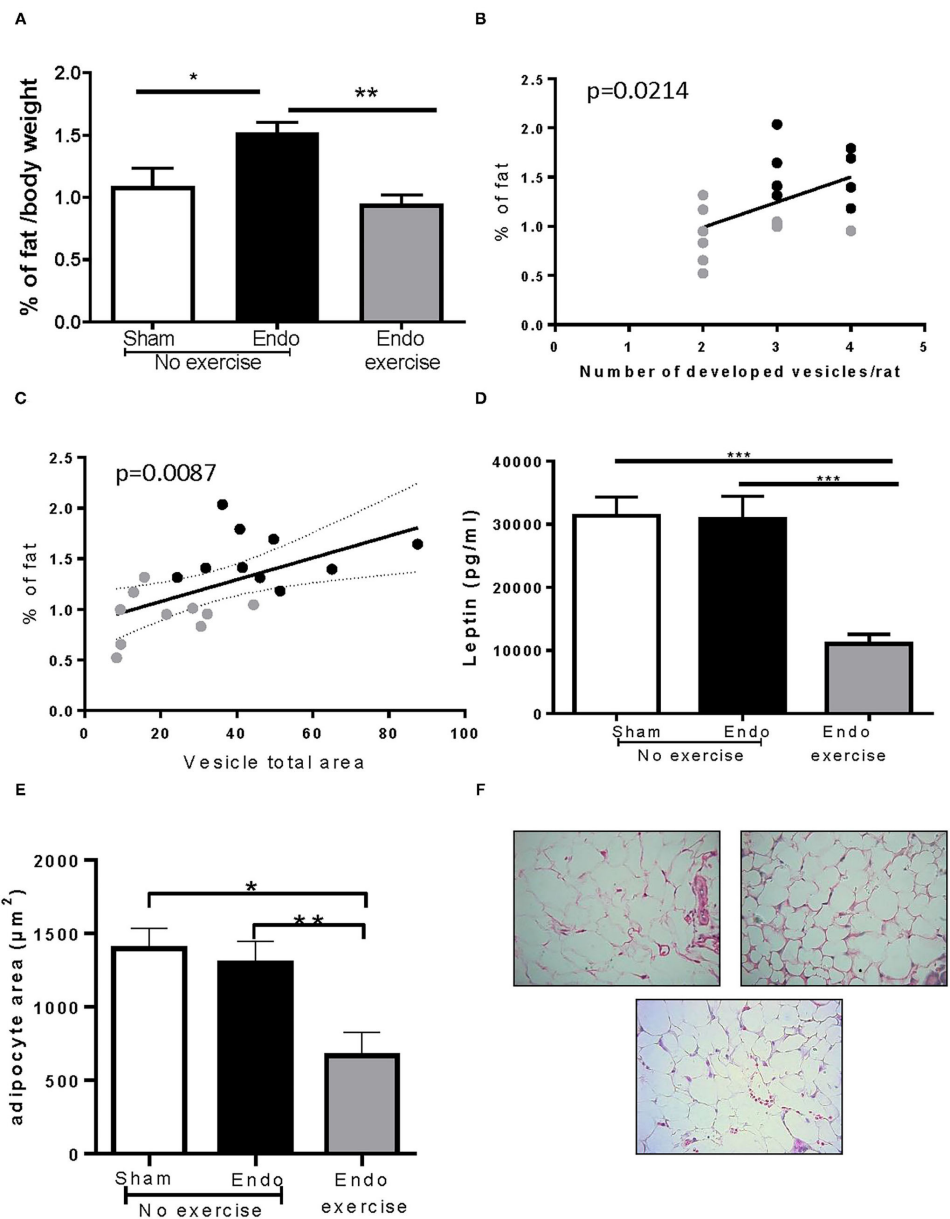
Effect of exercise on body fat. **(A)** Endo-Exercise animals had significantly less mesenteric fat when compared to Endo-No exercise; **(B)** Animals with higher % of fat developed more vesicles; **(C)** Animals with a higher % of fat had vesicles with a larger total area. **(D)** Endo-Exercise animals had significantly less circulating leptin in serum and **(E)** significantly smaller adipocytes. **(F)** Representative photos of adipocytes in mesenteric fat from (left to right): Sham-No exercise, Endo-No exercise and (bottom) Endo-Exercise animals, ×40 magnification (*n* = 9–10/group ± sem; ^*^p < 0.05, ^**^p < 0.01, ^***^p < 0.001; *n* = 5–6/group ± sem for adipocytes; black circles represent Endo-No exercise, gray circles represent Endo-Exercise).

Interestingly Endo-No exercise animals had significantly higher amounts of mesenteric fat when compared to the Sham-No exercise (*p* < 0.05) and this was mitigated in Endo-Exercise (*p* < 0.01; Figure 4A). Rats with a higher percent of fat developed more vesicles (*p* < 0.05; Figure 4B) which were of a greater size (*p* < 0.01; Figure 4C). Circulating serum levels of leptin, a hormone predominantly made by adipocytes, was significantly decreased by exercise (*p* < 0.001; Figure 4D). The size of the adipocytes in the mesenteric white adipose tissue from Endo-Exercise animals was approximately half of those found in either Sham-No exercise (*p* < 0.05) or Endo-No exercise (*p* < 0.01; Figures 4E,F).

### Exercise Effects on Cytokines

**Figure 5:**
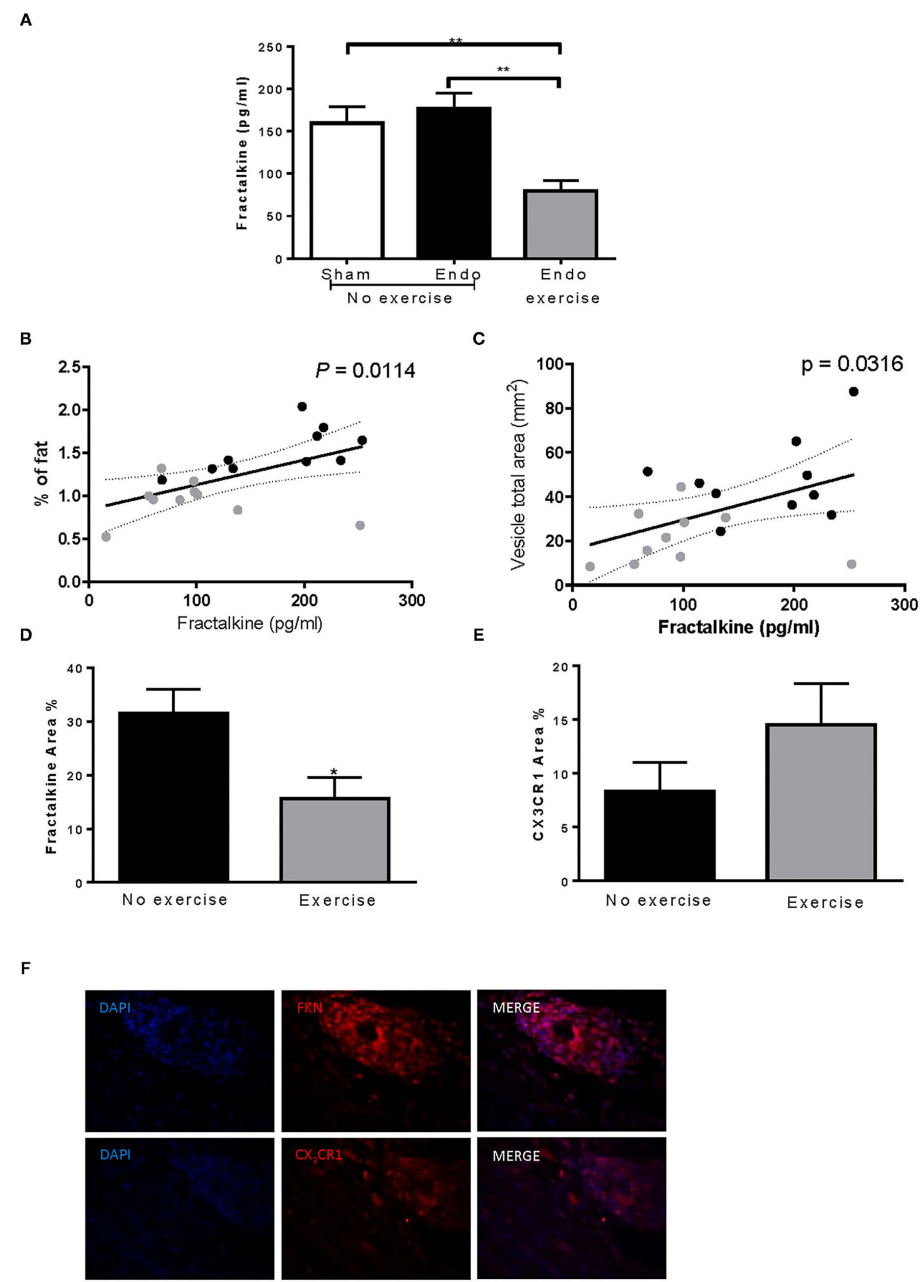
Exercise decreased fractalkine levels in endometriosis. **(A)** Exercise significantly reduced serum levels of fractalkine in endometriosis animals. Increased serum fractalkine levels in endometriosis animals correlated with **(B)** increased % fat and **(C)** larger vesicle size. **(D)** Fractalkine expression in vesicles from Endo-Exercise animals was significantly less compared to Endo-No Exercise. **(E)** CX3CR1 percent area almost doubled in Endo-Exercise but did not reach significance. **(F)** Representative immunofluorescence pictures of endometriotic tissue expressing fractalkine (FKN) or CXC_3_R1 (red), with nuclei counterstaining (DAPI; blue) at 100x magnification (*n* = 9–10 animals/group ± sem; ^*^p < 0.05, ^**^p < 0.01; black circles represent Endo-No exercise, gray circles represent Endo-Exercise).

Systemic levels of various cytokines, chemokines, and immunomodulators were assessed in serum collected at time of sacrifice (Table 1). Fractalkine, a chemokine known to be related with neuropathic pain, was downregulated by exercise (*p* < 0.01; Figure 5A). Lower levels of fractalkine in serum correlated with lower percentage of fat (Figure 5B) as well as decreased vesicle size (Figure 5C). Exercise also produced a significant reduction in levels of the chemokines RANTES (CCL5), which recruits leukocytes during inflammation, and LIX (CXCL5; Table 1).

**Table 1:** Cytokine levels in serum.

**Cytokines**	**Sham-no exercise**	**Endo-no exercise**	**Endo-exercise**	***p*-value**
Leptin	31,345 ± 2,963	30,746 ± 3,672	11,049 ± 1,492	<0.0001
IL-6	178.2 ± 29.44	191 ± 27.04	268.4 ± 71.86	0.4033
Fractalkine	159.6 ± 19.23	176.4 ± 19.17	79.84 ± 11.64	0.0012
IL-1	9.015 ± 0.6118	9.751 ± 0.4697	9.258 ± 1.413	0.8746
IP 10	755.8 ± 32.84	720.2 ± 33.14	639 ± 44.59	0.1074
MIP-1 alpha	34.41 ± 2.944	32.34 ± 1.808	29.09 ± 5.342	0.6122
TNF alpha	3.596 ± 0.4277	3.007 ± 0.377	3.827 ± 0.7934	0.5775
Eotaxin	16.01 ± 1.365	14.24 ± 1.395	14.65 ± 1.805	0.698
VEGF	166.2 ± 19.7	200.1 ± 21.85	134.1 ± 23.44	0.113
IL-10	48.94 ± 7.258	45.51 ± 5.935	42.22 ± 9.824	0.8344
RANTES	8,739 ± 1,112	7,510 ± 626.6	5,461 ± 840.1	0.0386
IL-1 alpha	16.44 ± 1.28	17.11 ± 2.575	12.42 ± 2.032	0.2305
IL-13	5.636 ± 0.5214	5.122 ± 0.3437	6.038 ± 1.067	0.687
IL-4	18.94 ± 2.24	19.24 ± 3.272	29.9 ± 7.497	0.2336
IL-5	286.8 ± 41.7	286.6 ± 29.16	398.7 ± 76.31	0.2607
IL-12-p70	34.04 ± 4.567	29.65 ± 6.424	60.48 ± 21.53	0.2538
IL-18	112.9 ± 18.31	151.9 ± 23.06	140.8 ± 40.5	0.6529
LIX	8,322 ± 767.6	7,273 ± 637.3	5,872 ± 485.2	0.0374
MCP-1	909.6 ± 92.08	1,010 ± 146.1	957.2 ± 97.3	0.8273
IFN	181.1 ± 36.69	135 ± 27.94	123.3 ± 48.76	0.5624
MIP-2	31.67 ± 1.786	34.67 ± 1.939	26.05 ± 3.966	0.1054
GCSF	10.57 ± 1.455	11.18 ± 2.527	10.99 ± 2.397	0.9811
IL-2	16.33 ± 3.065	14.72 ± 2.695	25.53 ± 13.7	0.6128
IL-17	26.57 ± 4.505	23.46 ± 2.926	36.65 ± 13.19	0.5334

*Values are presented as Mean ±SEM. Values are in pg/ml*.

Immunofluorescent staining for fractalkine expression in vesicles from Endo-Exercise animals was significantly less compared to Endo-No Exercise (*p* < 0.05; Figures 5D,F), while the expression of its receptor CX_3_CR1 almost doubled but did not reach significance (Figures 5E,F). No significant differences in fractalkine or its receptor were found in the mesenteric fat (data not shown).

**Figure 6:**
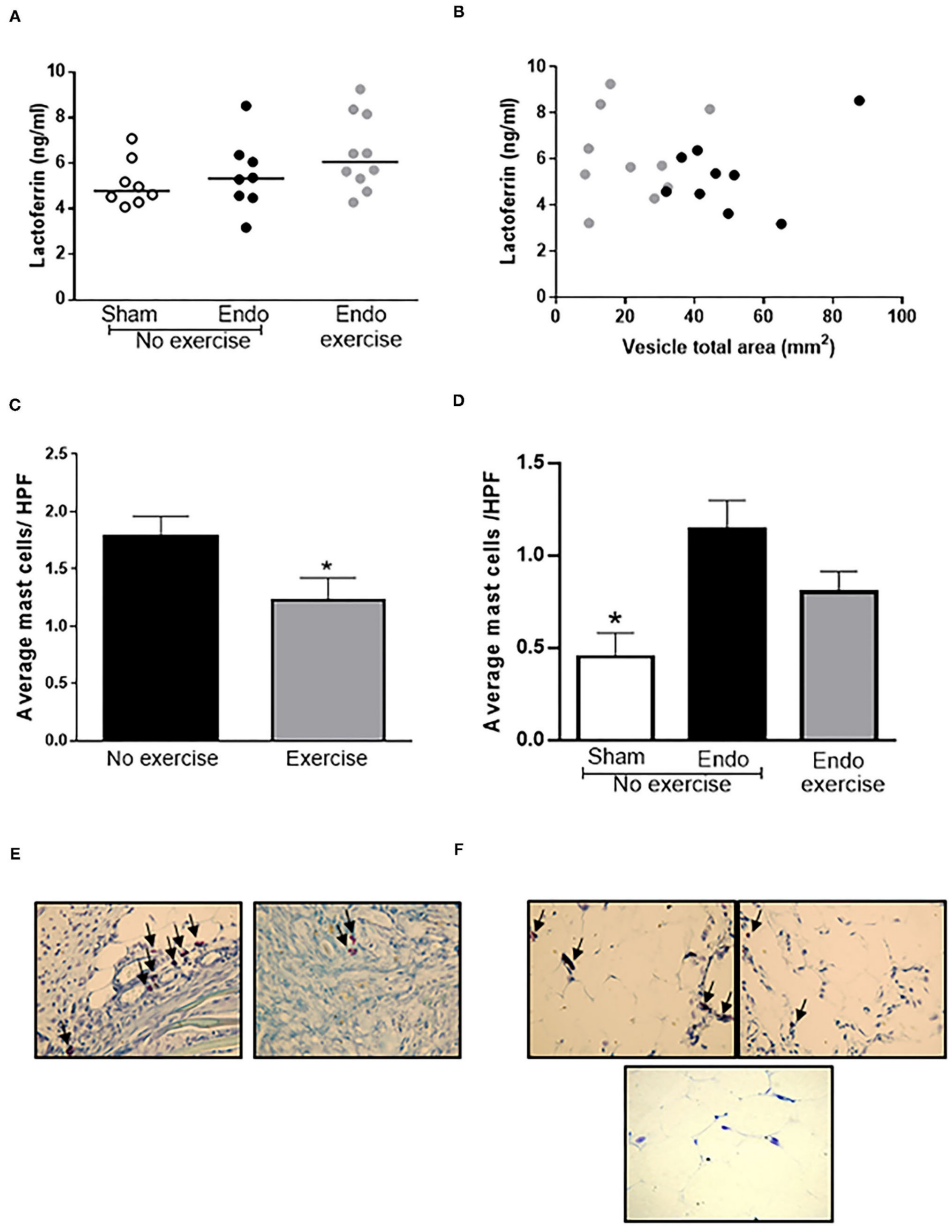
Exercise decreased mast cell infiltration. **(A)** Higher levels of lactoferrin were found in the peritoneal fluid of Endo-Exercise animals and **(B)** these tended to correlate with smaller vesicles. **(C)** Exercise resulted in a significant decrease in the number of mast cells found in vesicles, with **(D)** a similar pattern observed in the mesenteric fat. Representative photos of mast cells in **(E)** vesicles and **(F)** mesenteric fat from (left to right) Endo-No exercise and Endo-Exercise animals (bottom), Sham-No Exercise x40 magnification (*n* = 9–10 animals/group ± sem; ^*^p < 0.05 compared to Endo-No exercise; black circles represent Endo-No exercise, gray circles represent Endo-Exercise; arrows indicate mast cells).

In the peritoneal fluid, levels of lactoferrin, a natural immunomodulator, was found to be higher in exercised animals, and tended to inversely correlate with lesion size (*n* = 8–9/group; *r* = −0.1093) suggesting that it might be released to counteract the inflammation in the endometriosis animals (Figures 6A,B).

The average mast cell count decreased significantly in vesicles in Endo-Exercise (*p* < 0.05; Figures 6C,E) and was also lower in mesenteric fat (*p* = 0.06; Figures 6D,F).

### Exercise Modulated Estrogen Receptors in Vesicles and Mesenteric Fat

**Figure 7:**
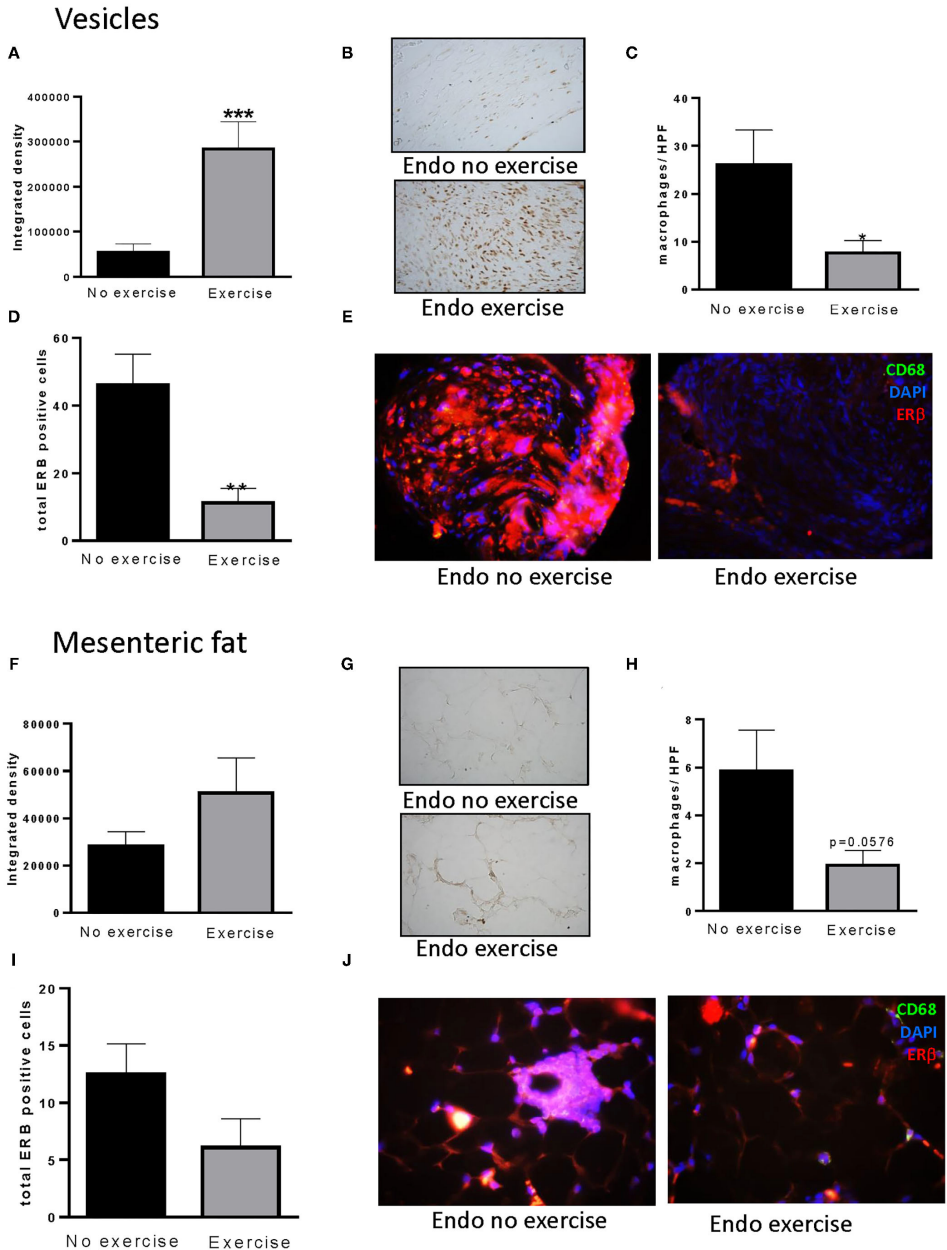
Exercise impacts estrogen alpha receptors in vesicles and mesenteric fat. **(A)** Increased levels of ERα were found in vesicles of Endo-Exercise animals. **(B)** Representative photos of immunohistochemical staining for ERα in vesicles, x100. **(C)** Exercise decreased the total number of macrophages found per high power field and **(D)** the number of ERβ positive cells in vesicles. **(E)** Representative photos of immunofluorescent staining of ERβ and macrophages (CD68) in vesicles, x100. **(F)** Similar patterns were observed for ERα in mesenteric fat adjacent to the vesicles. **(G)** Representative photos of immunohistochemical staining for ERα in mesenteric fat, x100. **(H)** Exercise decreased the total number of macrophages found per high power field in mesenteric fat, and **(I)** the number of ERβ positive cells. **(J)** Representative photos of immunofluorescent staining of ERβ and macrophages (CD68) in mesenteric fat from endometriosis animals with and without exercise, x100 (*n* = 9–10 animals/group ± sem; ^*^p < 0.05, ^**^p < 0.01, ^***^p < 0.001).

Although we observed no differences in serum levels of estradiol between treatment groups at time of sacrifice, nor variations with stage of cycle (data not shown), exercise resulted in a significant increase in the levels of ERα in the vesicles (*p* < 0.001: Figures 7A,B) concomitant with a reduction in macrophage infiltration (*p* < 0.05; Figure 7C) and ERβ immune-positive cells (*p* < 0.01: Figures 7D,E). This pattern was also noted in the adjacent mesenteric fat (Figures 7F–J). A positive correlation between ERβ and number of macrophages was found in vesicles (*p* < 0.01), with a tendency toward a similar pattern between ERβ and percent body fat observed in the mesenteric fat surrounding the vesicles (Supplementary Figure 3).

**Figure 8:**
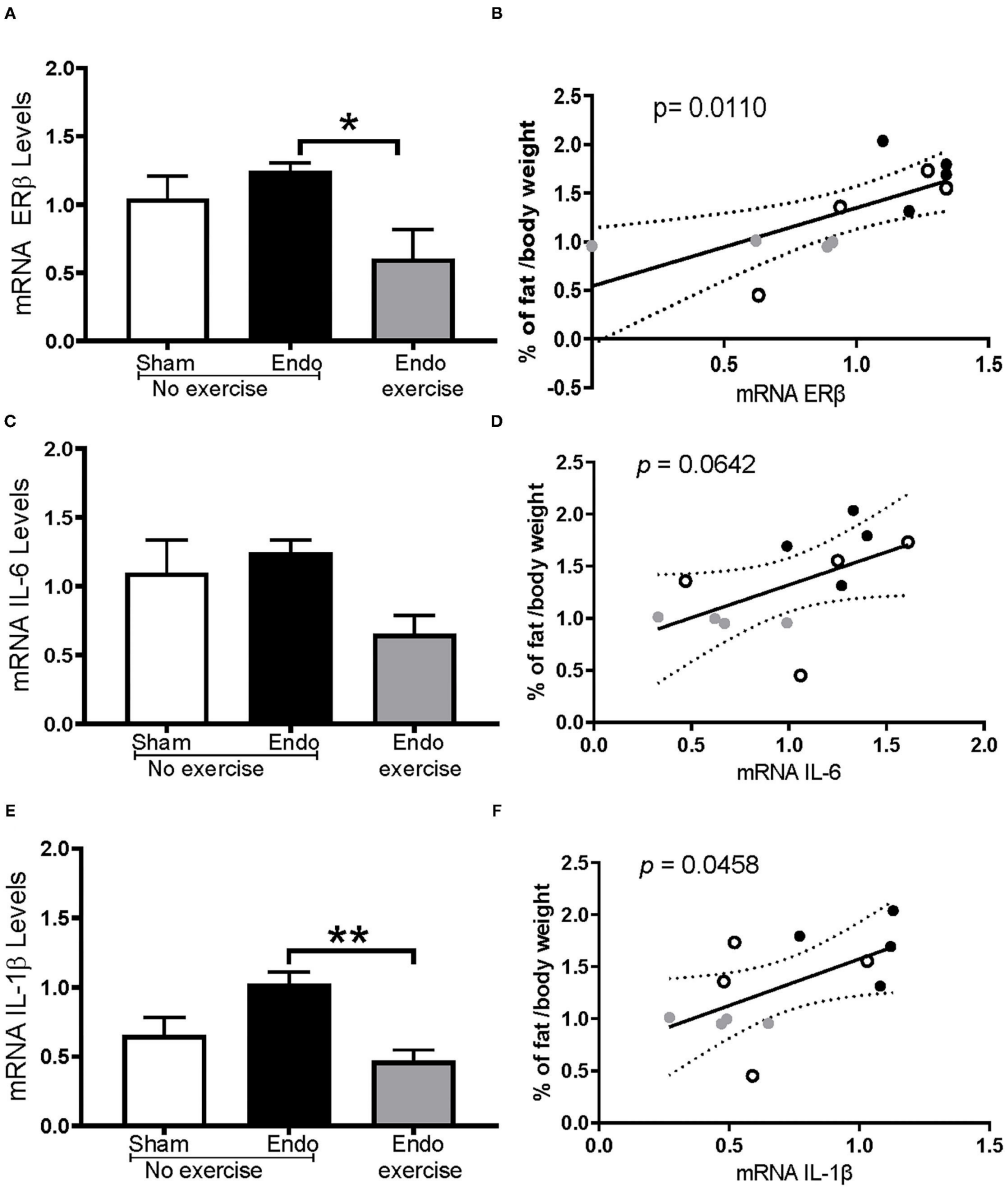
Exercise impacts estrogen receptor beta and inflammatory cytokines in mesenteric fat. **(A)** mRNA ERβ expression levels were significantly decreased in Endo-Exercise animals and **(B)** were significantly lower as the % of fat decreased. **(C)** Levels of IL-6 were mitigated by exercise and **(D)** these animals had a lower % of fat which correlated with a lower expression of IL-6 but didn't reach significance (*p* = 0.0642). **(E)** Endo-Exercise rats had a significantly lower expression of IL-1β which also **(F)** correlated with lower % of mesenteric fat. (*n* = 4/group ± sem; ^*^p < 0.05, ^**^p < 0.01; white circles represent Sham-No exercise, black circles represent Endo-No exercise, gray circles represent Endo-Exercise).

ERβ mRNA expression levels in mesenteric fat were also significantly decreased by exercise (*p* < 0.05; Figure 8A), in tandem with decreased levels of the inflammatory cytokines IL-6 (Figure 8C) and IL-1β (*p* < 0.01; Figure 8E). In each case these correlated with percent body fat reaching significance for ERβ (Figure 8B) and IL1β (Figure 8F) but not for IL-6 (Figure 8D). No significant differences were found in ERα expression.

## Discussion

Prior studies from our group and others have demonstrated that exposure to stress can modulate the HPA axis exacerbating the development and severity of endometriosis (17), and highlighted the impact and prevalence of psychological stress in endometriosis patients (22). Importantly, the level of stress controllability has been shown to modulate the pathophysiology of endometriosis, where those animals exposed to a “controllable stress” protocol had smaller lesions and less intestinal damage (21). These results provide impetus for investigations into the development of therapies targeted toward alleviating this condition in patients as reviewed in Appleyard et al. (23). Psychological stress and physical activity are thought to be reciprocally related, with significantly less unhealthy days reported in adults who exercise in line with current recommendations (24). In light of the fact that only a small percentage of adults in the United States meet the guidelines for physical activity, objectives under Healthy People 2030 include increasing the proportion of adults who carry out physical activity for health benefits and increasing engagement in physical activities by older adults with cognitive impairment (25, 26).

As mentioned earlier, the effects of physical activity and exercise in patients with endometriosis still require elucidation with very few studies conducted and little experimental data (12), although positive trends have suggested its potential benefit as a complementary treatment strategy (27). Patients with endometriosis who exercise scored better on mental health parameters of the SF-36 questionnaire (28), suggesting that social (distraction, interactions) as well as physical mechanisms (endorphin release) may be of benefit. Awad et al. (29) examined the impact of an 8-week exercise program (including postural correction, breathing, and treadmill walking) on women with mild or moderate endometriosis who had severe premenstrual pelvic pain and found significant reductions in pain but did not directly assess disease severity. Further, the outcomes of this study were confounded by concomitant administration of a hormone treatment with no control group. Moreover, a recent systematic review concluded that the effect of physical activity and exercise as treatments for endometriosis-symptoms could not be fully understood in patient studies to date due to significant limitations [design flaws, confounding factors, lack of controls, and relevant outcomes (30)].

Experimental studies on exercise in animal models of endometriosis are sparse and while subject to shortcomings they have provided invaluable insights into potential mechanisms mediating the beneficial effects of physical activity. Preliminary data from our lab demonstrated the beneficial impact of swim exercise on vesicle size and development, but were confounded by the fact that swimming is, by its nature, a form of stress (31). Likewise, Montenegro et al. (32) examined the impact of swim exercise varying in duration from 1 to 5 times a week also finding beneficial effects as evidenced by smaller lesion size. Other studies have shown beneficial effects of exercise but didn't consider variables such as amount and intensity of exercise, impact on food, fat levels, and hormones, thus limiting a deep understanding of the mechanisms involved. Our study helps fill this gap by showing associations with immune cell recruitment and changes in estrogen receptor expression that may underlie the benefits observed in exercised rats.

In this study we elected to use voluntary wheel running in an effort to more realistically capture the exercise consequences vs. forced treadmill use, which has been shown to produce negative physiological adaptations in the neuroendocrine and immune systems commonly associated with chronic stress such as adrenal hypertrophy (33–35). In rats, voluntary wheel running of 6 weeks duration prevented learned helplessness (36) and increases in fear conditioning produced by exposure to uncontrollable stress (15), while reducing adverse effects of CNS on depressive behaviors in rats (37). Voluntary wheel running can also attenuate neuropathic pain measured by Von Frey assessment of allodynia (16). As noted in prior studies, activity in the animals increased sharply during the acclimation period along with increased food consumption [reviewed by (38)]. Interestingly, those animals with higher cumulative distance developed significantly less vesicles, as did those with higher average speeds.

The mechanisms behind the potential beneficial effects of exercise in endometriosis are not completely understood, but our results point to a role of anti-inflammatory mechanisms and immune cell infiltration. The release of myokines by the exercising muscles have been postulated to act on distant organs including the adipose tissue, as well-production of anti-inflammatory cytokines (30, 39, 40). The chemokine CX_3_CL1, also known as fractalkine, as well as its receptor CX_3_CR1 have been documented to play a crucial role in neuropathic pain and inflammation (41), and enhance invasiveness of endometrial cells (42). CX_3_CR1 is known to be expressed in mast cells and macrophages (43). Fractalkine, which mediates leukocyte recruitment, has been shown to be secreted by adipose tissue indicating a possible pathway for the inflammation and pain processes in endometriosis. Our results suggest that voluntary exercise, *via* its impact on body fat and adipocyte size, can decrease the expression of fractalkine while increasing its receptor, regulating immune cell recruitment to the endometriotic lesions. In addition, through mast cell staining with toludine blue, we observed a significant reduction in the average number of mast cells per high power field in the Endo-Exercise group when compared to non-exercised animals suggesting decreased inflammation around the vesicles. Mast cells secrete cytokines and mediators which can chemoattract other inflammatory cells to the site, causing release of vasoactive amines that are associated to extravasation of cells into the affected tissue area, and induction of pain (44). Our prior work found higher levels of mast cell infiltration in endometriosis which was worsened by uncontrollable stress (45).

We also studied the effects of exercise on estrogen receptor expression in fat and vesicles. Estrogen plays an important role in the growth and persistence of endometriotic tissue along with inflammation [reviewed by (46)], and its effects are mediated by receptors found both intracellularly and in the plasma membrane of cells. In endometriosis patients, there is a dysregulation in estrogen receptor expression, where lesions show increased expression of the ERβ and reduced expression of ERα (47–49). ERβ has thus been suggested to be a therapeutic target (50). These receptors are also present in visceral fat and dysregulation of their function is known to correlate with increased adipose tissue (51). In the present study we observed the ability of exercise to shift this balance toward increased levels of ERα and decreased ERβ more similar to the scenario found in normal endometrium.

We observed increased mesenteric fat and serum leptin levels in the endometriosis animals that were decreased by exercise. Leptin, secreted by adipocytes, has been associated with angiogenesis and inflammation in endometriosis with circulating levels in patients found to be high or unchanged which seems to depend upon the location and severity of the disease (52, 53). The relationship between adiposity and endometriosis occurrence and severity is still not fully understood, in part due to the fact that BMI does not equate directly to either body composition or amount or distribution of adipose tissue (54–56). Associations between peripheral adipose tissue, and endometriosis have been shown [reviewed by (54)], suggesting the contribution of white adipose tissue to the pathogenesis of the disease. This biologically active tissue located in the peritoneal area was found to be greater in our endometriosis animals correlating with vesicle size. A relationship between adipose tissue and decreased muscle mass has also been observed (57). Although it is still unclear precisely how exercise might modulate estrogen receptor expression some data suggests that ERα is important for skeletal muscle fitness playing a critical role in mitochondrial biogenesis, which can help to increase fat oxidation and promote adaptive responses (58, 59). Our results suggest that voluntary exercise might protect against endometriosis and alleviate the associated symptomatology *via* modulation of estrogen receptors in vesicles and in adipose tissue.

While the results presented here represent an important first step toward understanding the impact of exercise in this condition there are some limitations. The choice of voluntary wheel running does not allow us to control the distance and intensity of the run for each animal, and our results suggest that these variables are indeed important for the beneficial effects. However, the variation observed here is representative of the natural range of animal activity and shows how that can translate to varying exercise regimes in the clinic. The changes in the mesenteric fat were predominantly focused on fat adjacent to the vesicles themselves and it is not known how those changes might be extrapolated to the adipose tissue and fat found at further locations, and this will be investigated further in follow up experiments. We are aware that exposure of the animals to the exercise prior to the induction of the endometriosis somewhat limits the applicability of these outcomes to use in the patient population. As such, ongoing studies by our group are now examining the impact of exercise as an intervention *after* the induction of endometriosis to represent the translatability of these outcomes more accurately to patients with this condition. Future studies aimed to evaluate the effects in the presence and absence of stress will provide additional mechanistic insight, as well as studies examining the effects of exercise on hyperalgesia.

In conclusion, our results demonstrate that voluntary physical activity ameliorates the development of vesicles and promotes decreased body fat composition in an animal model of endometriosis. The intensity of voluntary exercise also appears to contribute toward the degree of impact on this condition, with smaller vesicles noted in those animals running faster and longer distances. We hypothesize that the exercise is alleviating the associated symptomatology *via* immune modulation of the brain-gut axis and this will be explored in follow up analysis of the microflora, gut and brain. These results reinforce the potential beneficial effects of regular voluntary physical exercise as part of a daily routine to reduce endometriosis symptoms (pelvic pain, inflammation) in patients, and offers the potential for further exploration of this complementary therapy in the endometriosis patient population.

## Data Availability Statement

The raw data supporting the conclusions of this article will be made available by the authors, without undue reservation.

## Ethics Statement

The animal study was reviewed and approved by the Institutional Animal Care and Use Committee (IACUC) at Ponce Health Sciences University.

## Author Contributions

CA, IF, GC, and LA-N designed the study. MC, GC, JV-C, RR-M, JJ-G, and MM-C performed the experiments. CA, GC, MC, JV-C, JJ-G, LR, and MM-C analyzed the data. CA, GC, MC, LR, LA-N, and IF drafted the manuscript. All authors critically revised the manuscript for important intellectual content and have read and approved the final manuscript.

## Funding

These studies were supported by an Institutional Development Award (IDeA) from the National Institute of General Medical Sciences of the National Institutes of Health (NIH) under Grant Number P20 GM103475-16 and by NCCIH R15AT009915 and NIMHHD G12MD007579 (RCMI BRAIN Core). LR and MM-C were supported by training grants R25GM082406 and R25GM096955, respectively.

## Author Disclaimer

The contents are solely the responsibility of the authors and do not necessarily represent the official views of the NIH.

## Conflict of Interest

The authors declare that the research was conducted in the absence of any commercial or financial relationships that could be construed as a potential conflict of interest.

## Publisher's Note

All claims expressed in this article are solely those of the authors and do not necessarily represent those of their affiliated organizations, or those of the publisher, the editors and the reviewers. Any product that may be evaluated in this article, or claim that may be made by its manufacturer, is not guaranteed or endorsed by the publisher.
