# Supplementary material for: Voluntary Wheel Running Reduces Vesicle Development in an Endometriosis Animal Model Through Modulation of Immune Parameters
Source: Front Reprod Health. 2022 Jan 26;3:826541. doi: 10.3389/frph.2021.826541 (PMC9580825; doi:10.3389/frph.2021.826541)
Supplement: Supplementary file 1 [file Data_Sheet_1.PDF]

Supplementary Figure 1

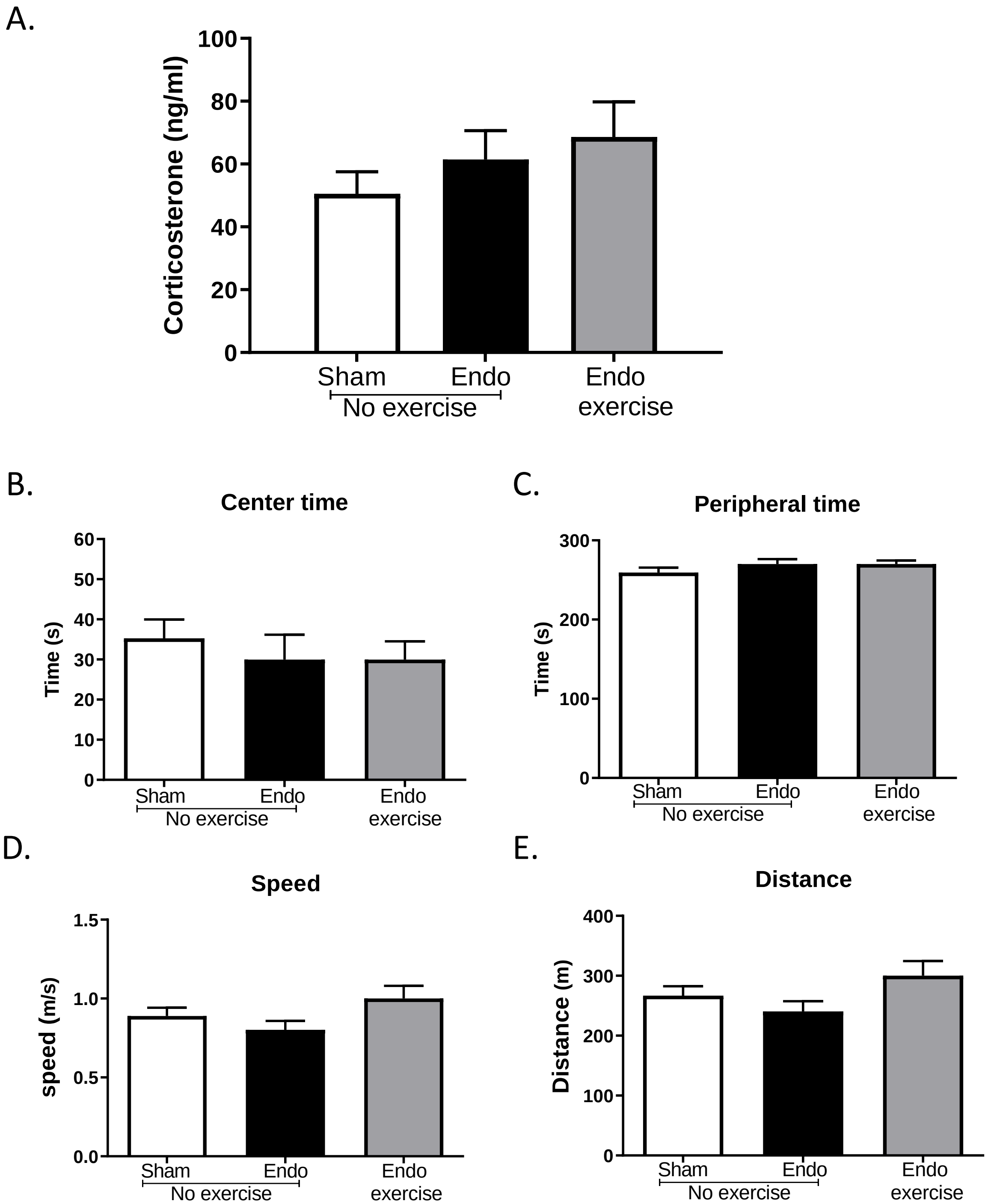

Supplementary Figure 1. *Effect of voluntary exercise on anxiety parameters*. (A) No significant differences in corticosterone levels were found between treatment groups. Time spent in the (B) center or (C) periphery in the open field behavioral test were not significantly different between groups prior to sacrifice. No differences were observed in (D) speed or (E) distance travelled. (n=9-10/group ± sem)

Supplementary Figure 2

A.

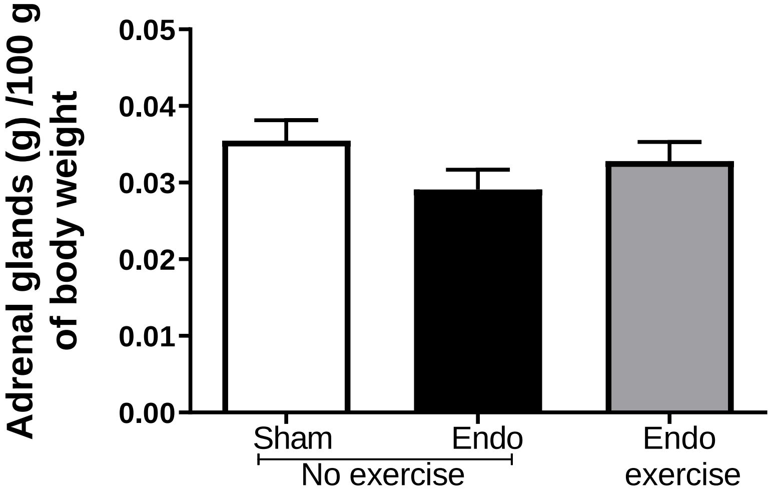

B.

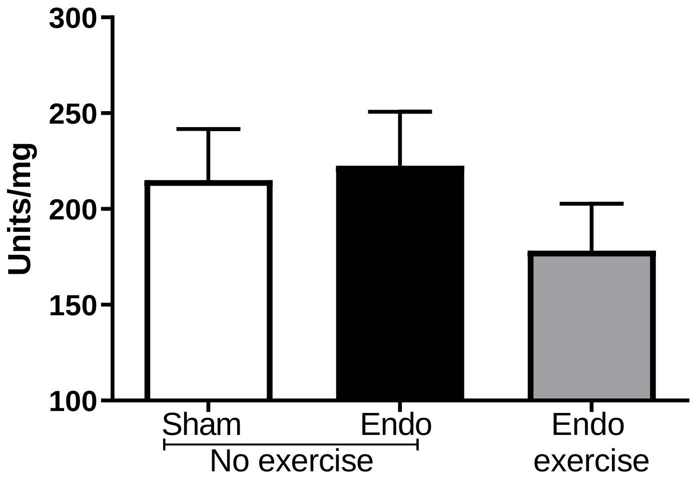

Supplementary Figure 2. *Effect of voluntary exercise on adrenal weight and uterine neutrophil infiltration*. (A) No significant differences were observed in the weight of the adrenal glands between treatment groups. (B) Neutrophil infiltration as measured by units of myeloperoxidase activity/mg of uterine tissue did not differ significantly between the treatment groups (n=9-10/group ± sem).

Supplementary Figure 3

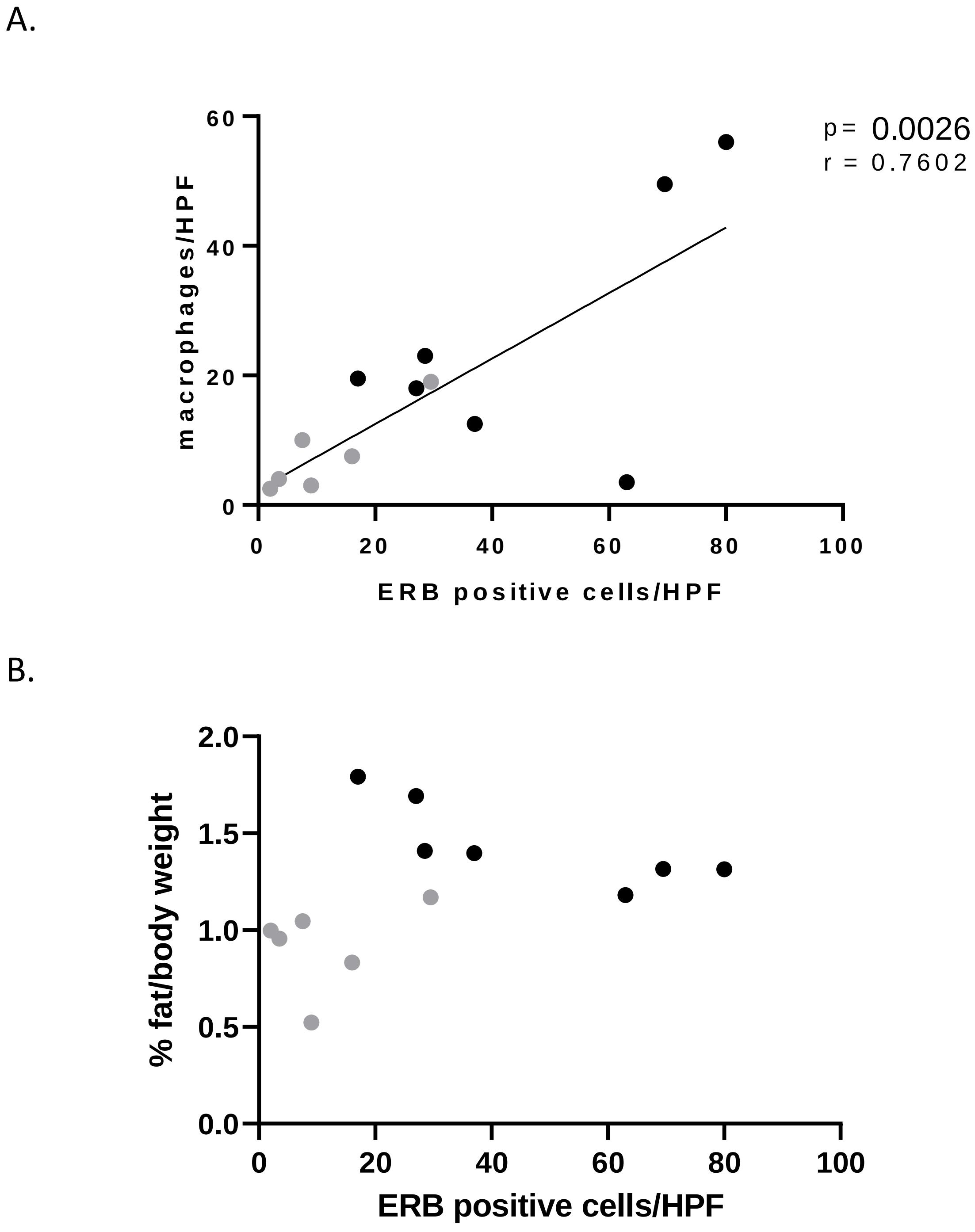

Supplementary Figure 3. *Correlations with Estrogen Receptor beta*. (A) A positive correlation between ERβ positive cells and number of macrophages was found in vesicles ($p<0.01$). (B) There was a tendency for more ERβ positive cells in the mesenteric fat adjacent to the vesicles with higher percentage of fat/body weight, but this did not reach significance (n=6-7/group; black circles represent Endo-No exercise, grey circles represent Endo-Exercise).
